# Some Scientific Aspects of Insanity

**Published:** 1892-01-16

**Authors:** 


### Some Scientific Aspects of Insanity.

### IX.—MANIA.

The opposite condition to melancholia is called mania, or mental exaltation. The essential distinction between mania and melancholia is that in the one there is mental exaltation—in the other mental depression. It is a very frequent thing to see mania and melancholia confounded with one another, the reason being that mental excitement, which is common to both mania and melancholia, is popularly supposed to be a quality only of mania. A melancholic patient may be excited, restless, and noisy, but depression in one of its forms is always present. There is the anxious countenance, the self-accusative delusion or the dread of impending danger. In mania it is entirely different. There is the attitude or the expression of great happiness, of superiority, of arrogance, or of antagonism. Therefore, the great distinction to bear in mind is that in melancholia there is present one or other of the symptoms which indicate mental depression, while in mania there is present either in the manner or the behaviour of the patient one or other of the symptoms which indicate exaltation of spirits.

When dealing with melancholia I stated that it was of all mental diseases the one least removed from sanity. Mania is a step more removed, for though a sane man may become very intensely depressed in spirits for a considerable period of time, yet a sane man cannot become mentally exalted for a correspondingly long period unless he is either drunk or insane.

When a person becomes maniacal there takes place in his mental condition a complete change of disposition. The sedate, retiring, modest man becomes talkative, boastful, self-asserting. He loses his regard for the feelings of others. He loses his finer power of judging correctly, and though there is often a superficial smartness manifested in attempts to pass disparagingly witty remarks, there is really intellectual decay or decline rather than power. If the disease advances, the patient becomes very restless, more talkative, sleeps very little, and exhausts a great deal of his energy. If there is a yet greater advance the patient becomes wildly incoherent, dances, gesticulates, and shouts. He appears to be unconscious of what goes on around him, or to be blindly furious and impulsive, directing his violence towards those with whom he comes into contact. These are briefly the mental symptoms. With the advent of the disease there is a great stimulus given to the bodily functions. If the patient is weakly, slow in his movements, flabby as to his muscles, pale and downcast in countenance, he at once becomes strong, the circulation becomes rapid, the eye glistens, the cheeks become slightly suffused, and the bodily actions become vigorous and animated. The patient becomes muscularly firmer, his appetite improves, his bowels come to move with great regularity, and there is a tendency to put on flesh. As the disease advances, and as the fire of nervous energy burns unabated, the body begins to manifest signs of failing, the tongue becomes furred, all superfluous fat disappears, the eyes become sunken and brighter, the bowels tend to operate oftener than once a day—the appetite is perhaps a little more capricious. With a still further advance of the disease there is a tendency towards bodily emaciation, the tongue becomes coated, the temperature rises, there is almost constant wakefulness, a moist, clammy skin, a tendency to refuse food, and symptoms of physical failure generally.

Like melancholia, mania is divided into several varieties. There is always present, however, the one fundamental distinctive feature—mental exaltation. 1st. The first variety is that which is called simple mania. *Simple Mania* is essentially a change in the character of an individual which is characterised by mild excitement, defective judgment, injudicious conduct, and extravagance in language and behaviour. It is frequently seen in asylums to occur at regular intervals in the mild attacks of insanity in the chronic insane. When a person of ordinary disposition assumes suddenly and without cause an extraordinary disposition, when he or she becomes unusually talkative, unnecessarily extravagant, irregular in habits, and in addition manifests a disregard for morality, even though to the eyes of a stranger there might appear nothing very extraordinary in the person's behaviour, yet to his intimate friends there is an evident alteration of character—that person may be said to labour under simple mania. The extravagance of conduct and of behaviour may become so marked as to make it necessary to deprive the individual of his liberty. When placed in asylums and otherwise under the care of doctors and attendants, these patients are often extremely troublesome. They devote their time and their energy to fault-finding and criticism of the institution in which they chance to be incarcerated. Owing to their intelligence, their disregard for the feeling of others, and their proclivity to work mischief, there is no class of the insane which can render the life of their guardians so miserable as patients labouring under simple mania.

2nd. The next variety of mania to be mentioned is *Acute mania*. In medicine a disease is called acute when it is of considerable severity, and when it runs a definite, more or less rapid course, generally ending in recovery. Now acute mania implies that the brain functions of the patient are intensely modified. Further, it implies that the disease will only last a certain time, and that it will end within a year either in death, chronic insanity, or recovery. In the great majority of instances it terminates in recovery.

In a typical case of acute mania the patient is noisy, unceasingly active, very talkative, and often incoherent in his conversation. With regard to his bodily symptoms, his temperature is usually high, especially at night, ranging from 99 to 101. His tongue is usually coated; his appetite may be voracious, but it is often capricious, owing to the fact that the patient is too much engrossed and busy to bestow thought upon such a serious matter as taking his meals. He is usually sleepless, and often noisy and destructive at night. As the disease continues there is a perceptible falling off in bodily condition, and despite the excitement, there is a fagged and pale expression of countenance. The patient requires the most constant vigilance on the part of the attendants to prevent him from injuring himself, from destroying property, and in order to secure cleanliness of habit.

